# Is Deep Hypothermic Cardiac Arrest Mandatory in Aortic Arch Surgeries?

**DOI:** 10.21470/1678-9741-2020-0465

**Published:** 2024-01-30

**Authors:** Jignesh Kothari, Ishan Gohil, Kinneresh Baria

**Affiliations:** 1 Department of Cardiovascular and Thoracic Surgery, U. N. Mehta Institute of Cardiology and Research Centre, Affiliated B.J. Medical College, New Civil Hospital Campus, Asarwa, Ahmedabad, Gujarat, India

**Keywords:** Thoracic Aorta, Catheterization, Deep Hypothermia Induced Circulatory Arrest, Neuroprotection, Aortic Diseases, Thoracic Aorta Dissection

## Abstract

Cannulation strategies in aortic arch surgeries are a matter of immense discussion. Majority of time deep hypothermic circulatory arrest (DHCA) is the way out, but it does come with its set of demerits. Here we demonstrate a case with aortic arch dissection dealt with dual cannulation strategy in axillary and femoral artery without need for DHCA and ensuring complete neuroprotection of brain and spinal cord without hinderance of time factor. Inception of new ideas like this may decrease the need for DHCA and hence its drawbacks, thus decreasing the morbidity and mortality associated.

## INTRODUCTION

**Table:** 

Abbreviations, Acronyms & Symbols	
CPB	= Cardiopulmonary bypass
CNS	= Central nervous system
CT	= Computed tomography
DHCA	= Deep hypothermic circulatory arrest
EEG	= Electroencephalography
NIRS	= Near-infrared spectroscopy
RTA	= Road traffic accident
SjO₂	= Jugular bulb oxygen saturation
SSEP	= Somatosensory evoked potential

Aortic arch surgeries are always on the talks due to its complexity and varied outcomes of different strategies applied. The choice of optimal cannulation technique for surgeries of ascending aorta and aortic arch is a matter of intense debate. The search for best cannulation strategy is paramount, as it directly relates to the morbidity and mortality of the patient.

The most used strategy for aortic arch surgeries is central cannulation and deep hypothermic cardiac arrest allowing the clean blood less ideal field to work in. But it does pose a serious potential threat of neurological damage. It has well proven that the same time of DHCA in different individuals leads to varied outcomes ranging from completely asymptomatic to stroke^[1]^. Thus, several centers have been trying different cannulation and cardiopulmonary bypass (CPB) strategies to establish optimal outcome of arch surgeries and, at the same time, decrease the morbidity and mortality, thus performing safe surgery^[2,3]^.

## CASE REPORT

**Fig. 1:**
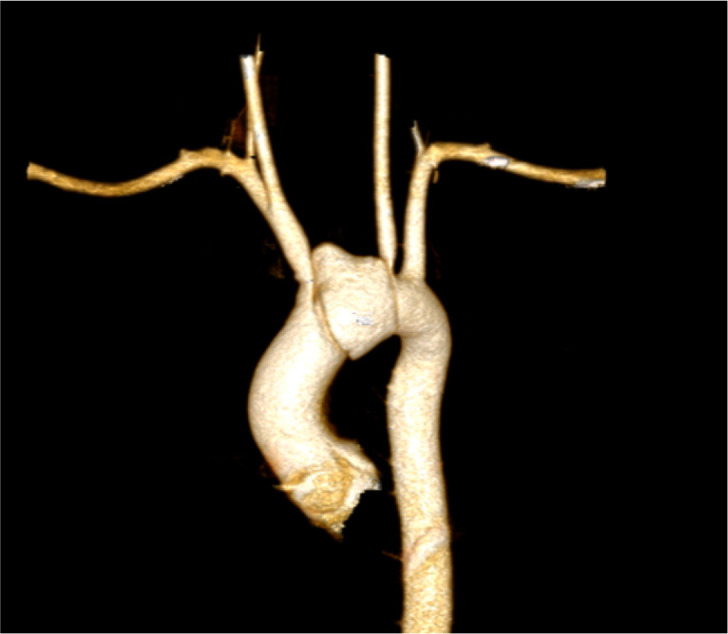
Preoperative CT scan (VRT reconstruction).

**Fig. 2:**
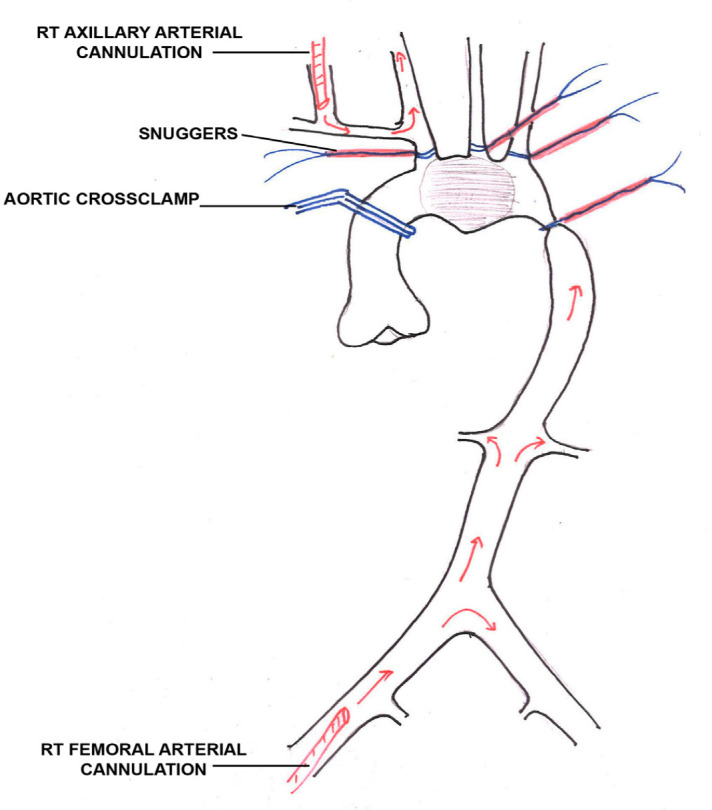
Cannulation strategy.

A 16-year-old male patient came with a history of road traffic accident (RTA) 2 days back and chest pain. On further investigation, patient was found to have aortic arch dissection between the origin of right brachiocephalic and left common carotid arteries causing stenosis of origin of both vessels (Figure 1). The patient had no family history of aortitis or any collagen vascular disorders. The patient was taken to the operation theatre and a unique cannulation strategy was applied. First, the right axillary artery was dissected and looped in the infraclavicular region. An 8-mm Dacron graft was anastomosed in an end-to-side manner using Prolene 6-0 continuous sutures. Axillary cannulation was done through this graft. Another arterial cannulation was done through the right femoral artery in routine manner. Hence dual arterial cannulation was done (Figure 2). Sternotomy was done and venous cannulation was performed through two-stage right atrial cannula for best drainage.

**Fig. 3:**
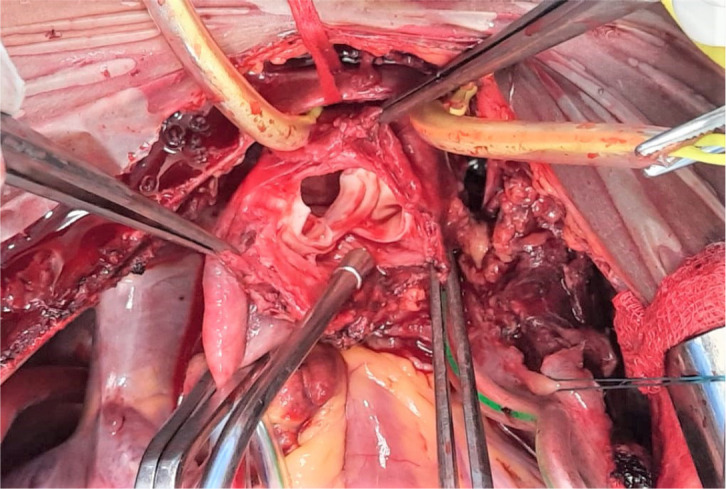
Intraoperative picture showing dissection flap between right brachiocephalic and left common carotid arteries.

The patient was taken on CPB and mild hypothermia using the dual arterial cannulation and two-stage right atrial cannulation to divide equal flows between both the arterial cannulas. All three great vessels in the arch were dissected and doubly looped to obstruct the lumen. The heart was arrested with aortic root cardioplegia. Proximal clamp was applied just before the junction of brachiocephalic trunk with the aortic arch and distal clamp was applied at the end of arch. With all the looped great vessels occluded, the dissected arch region was opened to discover that there was circumferential tear of intima of the arch in the region between brachiocephalic and left common carotid arteries (Figure 3). The arch was dissected to detach both halves as to make a proximal and a distal part of the arch. In each part the intima, media and adventitia were buttressed together using a Teflon felt with continuous Prolene sutures. These two halves were then anastomosed with each other in an end-to-end fashion using continuous suturing technique.

Deairing was done by applying aortic cross-clamp proximal to the aortic root vent cannula. Sequential opening of first the proximal arch clamp, the distal arch clamp and then the arch vessels was done to ensure adequate deairing of the complete arterial system. CPB is eventually weaned off and decannulation done as per routine protocol.

**Fig. 4:**
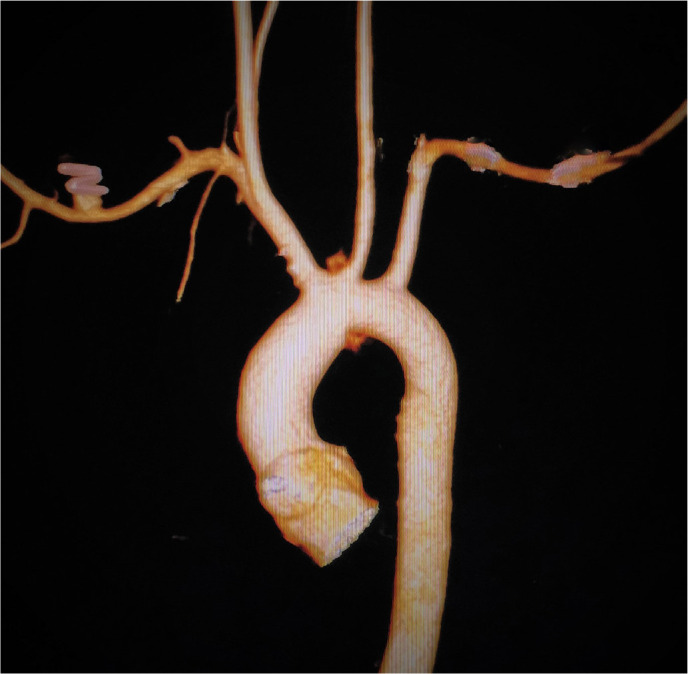
Postoperative CT scan showing almost normal arch with well-flowing branches.

The patient had an uneventful postoperative period, without any major or minor neurological deficit. Postoperative computed tomography (CT) angiogram showed normal caliber arch without any stenosis with good flow in all great vessels (Figure 4).

## DISCUSSION

DHCA is a technique employed to facilitate complete cardiovascular surgery. Complete cardiopulmonary arrest is induced to allow surgery on major blood vessels that cannot be bypassed intraoperatively and, therefore, upon which surgery would normally cause disruption to distal blood flow and profound hemorrhage in surgical field. Hypothermia applied causes depression of the cellular metabolism, thus protecting organs from ischemia, but central nervous system (CNS) is very volatile and very prone to deleterious effects of ischemia^[1]^. Thus, during DHCA, various strategies are applied for monitoring the CNS, like electroencephalography (EEG), somatosensory evoked potential (SSEP), near-infrared spectroscopy (NIRS), jugular bulb oxygen saturation (SjO₂) etc. Additionally, pharmacological neuroprotection is also done along with it. One of the prime factors in DHCA is time^[6]^. Ischemic time is of utmost importance hence the surgery is to be done hastily, which again at times increases the chances of complication.

All these above drawbacks of DHCA can be avoided by this dual cannulation strategy. Continuous antegrade cerebral blood flow is initiated, which ensures the neuroprotection. Various neuromonitoring devices can be avoided. The perfusion of other vital organs and lower limbs can be ensured through femoral cannulation^[2,5]^. Thus, the whole body is adequately perfused throughout the CPB, while ensuring a clear and bloodless field for surgery. In addition, this removes the critical time constraint associated with DHCA, thus less mishaps. The patient has a smoother postoperative course owing to the lack of ischemia, therefore, the morbidity decreases.

Arch surgeries are done till date in DHCA in almost every center in the world^[4]^. Here, we are trying to introduce the idea that DHCA might not be the only option serving this purpose. If there is a safer and equally effective option, then it should be surely explored and taken advantage of. This dual cannulation technique removes the hazards of DHCA while giving a perfect surgical field for anastomosis without critical time constraints.

## CONCLUSION

Cannulation strategy represents a critical choice that may play a crucial role in determining operative outcomes in aortic surgery. Although it takes time for new evidence in the literature to translate into common practice, there is no doubt that, in most centres, worldwide different strategies are rapidly emerging for both acute and chronic cases of aortic arch. This dual cannulation strategy for arch surgery is revolutionary to serve its purpose by providing proper antegrade cerebral flow along with distal body perfusion at the same time providing a great surgical field to work on. Techniques like these should be adopted and explored in different scenarios for their optimal usage.

**Table:** 

Authors’ Roles & Responsibilities	
JK	Substantial contributions to the conception or design of the work; or the acquisition, analysis, or interpretation of data for the work; drafting the work or revising it critically for important intellectual content; final approval of the version to be published
IG	Agreement to be accountable for all aspects of the work in ensuring that questions related to the accuracy or integrity of any part of the work are appropriately investigated and resolved; final approval of the version to be published
KB	Drafting the work or revising it critically for important intellectual content; final approval of the version to be published
